# Marjolin’s ulcer arising in pilonidal disease: a case report and historical perspective

**DOI:** 10.1093/jscr/rjaf964

**Published:** 2025-12-28

**Authors:** Paige Curcio, Frank Ross

**Affiliations:** NYU Grossman School of Medicine, 550 1st Ave, New York, NY 10016, United States; Department of Surgery, NYU Grossman School of Medicine, 550 1st Ave, New York, NY 10016, United States; Kimmel Hyperbaric and Advanced Wound Healing Center, NYU Langone Health, 240 E 38th St, New York, NY 10016, United States

**Keywords:** Marjolin’s ulcer, pilonidal disease, squamous cell carcinoma, biopsy, chronic wounds, nonhealing ulcers

## Abstract

We report a case of malignant transformation in a young woman with pilonidal disease—a rare manifestation of ‘Marjolin’s ulcer’. The patient initially presented to an outside hospital emergency department with an ulcer of the left buttock. Her family reported a longstanding mass present at this site prior to rupture. The patient had two ED visits and a surgical referral that did not result in biopsy, contributing to delayed diagnosis. She eventually presented to our wound clinic with a full-thickness, friable, frond-like ulcer with probable sacral extension. Immediate biopsy was performed and confirmed squamous cell carcinoma (SCC). Imaging revealed a buttock mass with lymphadenopathy. Fine-needle aspiration confirmed metastatic SCC in the inguinal lymph nodes. The disease was deemed unresectable and systemic therapy was begun. This case emphasizes the importance of biopsy in nonhealing ulcers, underscores the need for system-level processes to ensure follow-up, and demonstrates the enduring relevance of Marjolin’s original 19th-century observations.

## Introduction

Transformation of pilonidal sinus disease to squamous cell carcinoma (SCC) is exceptionally rare, occurring in ~0.1% of cases [1]. Between 1900 and 2022, 140 cases of carcinoma associated with pilonidal sinus disease have been documented [2], with cases in young, female patients being especially uncommon [1]. Reported outcomes are often poor, particularly when diagnosis is delayed or when regional metastasis is present. This case highlights the malignant potential of longstanding pilonidal disease, situates the findings within the original 1828 historical description by Jean Nicolas Marjolin of what is now known as Marjolin’s ulcer [3], and reviews current consensus on the timing and indications for biopsy of nonhealing ulcers.

## Case report

The patient is a 38-year-old female with a past medical history of Down syndrome and congenital heart disease, who initially presented to the emergency department of an outside hospital in the fall of 2024 with a 3 cm, draining ulcer in the upper left buttock. Her family reported a longstanding mass present at this site for over 15 years described as ‘golf-ball sized’ prior to rupture in the summer of 2024, after which the wound never fully healed. She was referred from the ED to a surgery clinic, but there was no record of her being seen in clinic. The patient re-presented to the ED 4 months later with persistent drainage from the ulcer and wound photographs that showed interval enlargement. She was seen by surgery who documented a history of prior pilonidal disease and referred the patient to wound care. The patient’s two ED visits and surgical referral did not result in biopsy at this stage, contributing to delayed diagnosis.

The patient was subsequently evaluated at our wound clinic in the spring of 2025. Examination revealed a vertically oriented, fissure-like, full thickness ulcer measuring 6 × 2.5 cm, with an estimated depth of 3–4 cm and probable extension to the sacral bone. The margins were irregular and frond-like, with friable easily bleeding tissue. A wedge biopsy was performed at this first visit. The family was instructed to pack the wound with dilute sodium hypochlorite-soaked gauze and cover with a foam dressing. Home nursing was arranged for dressing changes, and a CT abdomen/pelvis was ordered.

Histopathology demonstrated squamous cell carcinoma infiltrating the reticular dermis, and she was referred to surgical oncology. At the patient’s initial oncology visit additional symptoms of pain, pruritus, unintentional weight loss, and fatigue were documented. The CT abdomen/pelvis showed an 8.4 × 4 × 3.7 cm left buttock mass with suspicious right inguinal and bilateral internal iliac lymph nodes. Fine-needle aspiration of the inguinal nodes confirmed metastatic SCC. Given the extent of regional nodal disease, the case was deemed unresectable, and the patient was diagnosed with stage III cutaneous SCC. In the summer of 2025, she initiated systemic therapy with the PD-1 inhibitor cemiplimab. Currently, she continues immunotherapy with ongoing wound care and social work support.

## Discussion

**Figure 1:**
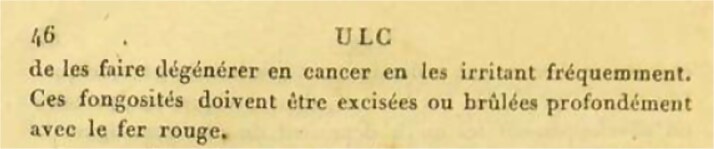
Excerpt from Ulcère. Translation: ‘These fungating growths must be excised or deeply burned with red-hot fire.’

**Figure 2:**
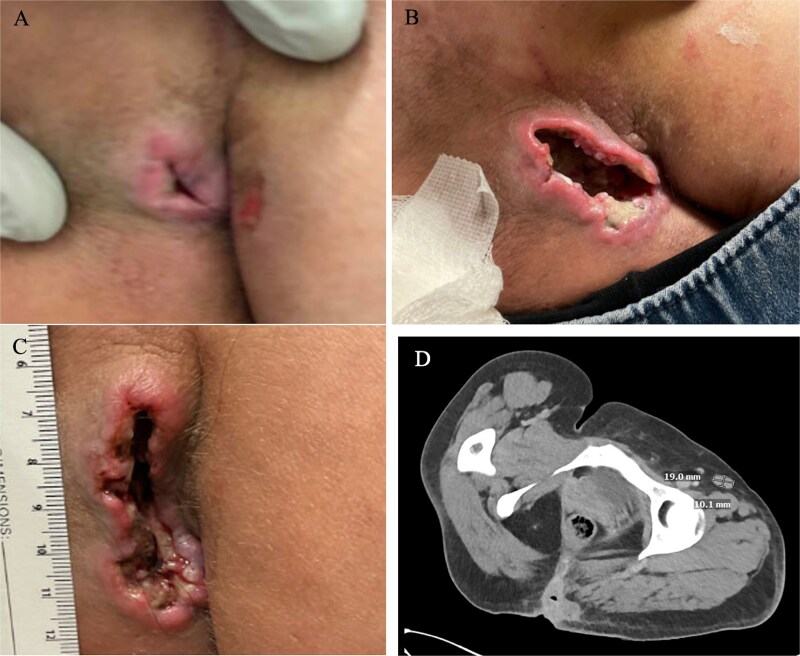
A 38-year-old female presenting with a nonhealing ulcer. (A) Initial presentation (3 cm). (B) Second presentation. (C) Presentation to wound care facility with a 6 × 2.5 × 3–4 cm wound. (D) CT abdomen pelvis. CT findings include an 8.4 × 4 × 3.7 cm left buttock mass with suspicious right inguinal and bilateral internal iliac lymph nodes.

Marjolin’s ulcer describes malignant transformation arising in chronic wounds, a phenomenon first characterized by Jean Nicolas Marjolin in his 1828 paper *Ulcère*. He noted that fungating, friable ulcers with villous projections, which he called the ‘warty ulcer’, ‘must be excised or deeply burned with a red-hot iron’ (*ces fongosités doivent être excisées ou brûlées profondément avec le fer rouge*) (Fig. 1) [3]. His early recognition that chronic irritation could provoke cancer underscores an enduring principle in wound care: vigilance is required when evaluating nonhealing ulcers. The lesion in the present case—friable, irregular, and slow to heal—was strikingly reminiscent of Marjolin’s original description nearly two centuries ago (Fig. 2).

Despite this long-standing awareness, diagnostic delay remains common. In this case, the patient had at least two earlier encounters where biopsy could have been considered. This delay likely contributed to progression of her disease. Current consensus guidelines recommend a low threshold for biopsy in non-healing wounds. Biopsy is advised for ulcers that fail to demonstrate improvement after 4 to 6 weeks of standard therapy or sooner when atypical features are present, such as abnormal granulation tissue, irregular margins, or unusual locations [4]. Prospective studies also support systematic biopsy of ulcers persisting beyond three months despite appropriate care, given the high incidence of malignancy detected under these circumstances [5]. Of note, the typical latency period for malignant transformation of a chronic wound is often measured in decades, with a mean latency of ~25 years [6].

Clinical studies have identified features associated with malignant transformation in chronic ulcers, including older patient age, atypical granulation tissue that is excessive at the edges, dull pink wound bases, smaller wound surface area, and duration of over 24 weeks [4, 7]. Earlier recognition and biopsy in this patient may have allowed for diagnosis at a surgically resectable stage. Treatment of SCC arising in pilonidal disease is particularly challenging once nodal involvement is present. This reality highlights the importance of addressing pilonidal disease early, since chronic or untreated disease fosters a cycle of inflammation and infection that increases the risk of malignant degeneration. Surgical excision of pilonidal disease before malignant transformation, or timely biopsy of nonhealing ulcers in this setting, is critical to preventing advanced and unresectable cancer.

Checkpoint inhibitors such as cemiplimab have emerged as systemic options for unresectable cutaneous SCC, including rare cases arising in pilonidal disease. A recent case series described partial responses to cemiplimab, though further research is needed to clarify overall outcomes of this therapy [8]. For this patient, initiation of cemiplimab offers hope for disease control, but the prognosis remains uncertain.

## Conclusion

This case underscores the importance of system-level processes that facilitate follow-up for patients referred from the ED with nonhealing wounds to ensure timely diagnostic evaluation. Efforts to prevent the loss of patients in transition between services are essential to avoid delayed diagnosis of malignant transformation. In summary, this case expands the limited literature on Marjolin’s ulcer arising from pilonidal disease, reinforces the critical role of early biopsy in nonhealing wounds, and demonstrates the enduring relevance of Marjolin’s 19th-century observations to modern surgical oncology.
